# Spatiotemporal and kinematic gait characteristics in older patients with type 2 diabetes mellitus with and without sarcopenia

**DOI:** 10.1038/s41598-025-00205-0

**Published:** 2025-05-27

**Authors:** Tomotaka Manabe, Wakako Tsuchida, Toshihiro Kobayashi, Masahiro Fujimoto, Takuma Inai, Kohei Kido, Shoma Kudo, Kensaku Fukunaga, Takanobu Saheki, Takafumi Yoshimura, Hitomi Imachi, Koji Murao

**Affiliations:** 1 Department of Endocrinology and Metabolism, Faculty of Medicine, Kagawa University, 1750-1 Ikenobe, Miki-cho, Kita-gun, Kagawa, 761-0793 Japan; 2 Division of Rehabilitation, Department of Medical Technology, Kagawa University Hospital, 1750-1 Ikenobe, Miki-cho, Kita-gun, Kagawa, 761-0793 Japan; 3 Integrated Research Center for Self-Care Technology , National Institute of Advanced Industrial Science and Technology (AIST), 2217-14 Hayashi-cho, Takamatsu, Kagawa, 761-0395 Japan; 4 Health and Medical Research Institute, National Institute of Advanced Industrial Science and Technology (AIST), 2217-14 Hayashi-cho, Takamatsu, Kagawa 761-0395 Japan

**Keywords:** Gait analysis, Kinematic parameter, Older patients, Range of motion, Sarcopenia, Type 2 diabetes, Type 2 diabetes, Ageing, Predictive markers

## Abstract

To investigate the differences in gait characteristics between older patients with type 2 diabetes with and without sarcopenia, using a three-dimensional motion analysis system. This cross-sectional study included 38 patients with type 2 diabetes aged ≥ 65 years and capable of independently performing activities of daily living. Participants’ demographic data, spatiotemporal gait parameters, and ranges of motion of the pelvis, hip, knee, and ankle joints during a gait cycle were collected. The participants were classified into sarcopenic (*n* = 15) and non-sarcopenic (*n* = 23) groups and compared. Significant reductions in walking speed, shorter stride lengths, longer stride time, stance and double support times, and decreased cadence were observed in participants with sarcopenia. In addition, participants with sarcopenia exhibited a significantly smaller range of motion for ankle joint plantar dorsiflexion. In patients with type 2 diabetes, gait characteristics differ depending on the presence or absence of sarcopenia. These findings may contribute to the development of new qualitative assessment methods to facilitate the timely detection of sarcopenia in this patient population.

**Supplementary Information:**

The online version contains supplementary material available at 10.1038/s41598-025-00205-0.

## Introduction

The global prevalence of type 2 diabetes is predicted to reach 780 million by 2045^1^. The development of type 2 diabetes is linked to increased mortality, complications such as sarcopenia, and an increased risk of falls^2–6^. Early detection of functional abnormalities, particularly gait issues, is crucial for improving diagnostic and therapeutic approaches. The prevalence of sarcopenia among older individuals in Asia is 7–12%^7^, whereas that among their counterparts with type 2 diabetes is 12–20%^2^. Glycemic control is influenced by a multitude of factors, including insulin secretion from pancreatic beta-cells, insulin sensitivity in peripheral tissues, such as the liver and skeletal muscles, cognitive function, as well as lifestyle factors such as diet and physical activity. Moreover, increasing attention focuses on the impact of conditions, such as sarcopenia and frailty, which are states of physical weakness, on worsening glycemic control. By the age of 80 years, humans lose approximately 40% of their skeletal muscle mass compared with when they were in their 20s^8^. In addition to age-related changes, poor nutrition, decreased physical activity, and chronic conditions, including diabetes, contribute to the development of sarcopenia^9,10^.

Skeletal muscle facilitates over 80% of glucose uptake in the periphery in response to insulin. This glucose is utilized as energy or stored as glycogen. Consequently, the decrease in skeletal muscle mass due to sarcopenia leads to reduced capacity for glucose metabolism in response to insulin. Additionally, the concentration of glucose transporter type 4, which is regulated by insulin and responsible for cellular glucose transport, decreases within skeletal muscle cells with age^11^. Therefore, insulin resistance worsens in patients with sarcopenia and type 2 diabetes and increases the risk of deteriorating glycemic control. The diagnosis of sarcopenia differs across regions and ethnicities, with various criteria being used worldwide. In Asia, the criteria proposed by the Asian Working Group for Sarcopenia (AWGS) are commonly used^7^. However, these diagnostic criteria for sarcopenia typically require specialized equipment and facilities for estimating skeletal muscle mass through techniques, such as bioelectrical impedance analysis (BIA) and grip strength and walking speed measurements. Meeting these requirements can be challenging in clinical settings, hindering the timely diagnosis of sarcopenia and the initiation of appropriate treatment. Consequently, a growing need exists to develop simpler evaluation methods.

Sarcopenia is a condition characterized by an age-related decline in skeletal muscle mass combined with decreased muscle function (strength or physical performance)^7,9^. Current diagnostic criteria primarily evaluate physical function through walking speed and grip strength, neglecting other body movement assessments. In recent years, measuring joint angles while walking from footage captured using a monocular camera has become possible. Understanding the gait characteristics of patients with type 2 diabetes and sarcopenia could underpin the use of artificial intelligence (AI) to detect the presence of sarcopenia in these patients from easily measurable joint angle data during walking. However, to our knowledge, no reports have addressed the differences in gait characteristics in patients with type 2 diabetes with or without sarcopenia. Mori et al.^12^ reported that older adult Japanese women with sarcopenia exhibited significant decreases in walking speed and stride length compared with healthy controls. However, studies evaluating the spatiotemporal parameters used to assess gait characteristics of individuals with sarcopenia are limited. Walking can be expressed using various parameters beyond walking speed, including cadence, step length, step width, stance time, swing time, and double support time. Analyzing kinematic features of walking, such as joint movements and postural changes, alongside spatiotemporal gait parameters, may provide fundamental insights for early detection of sarcopenia. Understanding differences in gait characteristics associated with sarcopenia is essential for establishing a simple measurement method for the early detection of the condition. Three-dimensional (3D) motion analysis systems involve attaching reflective markers to landmarks on the body and analyzing the raw data captured by multiple synchronized cameras to illustrate joint movements during activities, such as walking.

Therefore, in this study, we aimed to investigate the differences in gait characteristics between older patients with type 2 diabetes, with and without sarcopenia, using a 3D motion analysis system.

## Methods

### Research design

This was a cross-sectional study (no. UMIN 000054792, www.umin.ac.jp/ctr/). Patients with type 2 diabetes attending the Kagawa University Hospital who were aged ≥ 65 years and capable of independently performing activities of daily living were recruited for this study. Participants were excluded if they: (1) experienced frequent hypoglycemia; (2) had preexisting gait dysfunction due to cerebrovascular or neurological diseases; (3) participated in other clinical trials; (4) had severe renal dysfunction (serum creatinine level of ≥ 1.5 mg/dL); (5) had severe diabetic neuropathy; (6) had other serious complications; and/or (7) regularly engaged in competitive sports on a professional level. All participants were capable of walking independently and had normal or corrected-to-normal vision. The sample size was determined using G*Power software (version 3.1.9.6, Dusseldorf, Germany) to obtain sarcopenic and non-sarcopenic data based on a previous study^13^ and detect walking function. The effect size was 1.06, with an α level set at 0.05, power set at 0.80, and allocation rate of non-sarcopenia/sarcopenia set at 2.0. The Mann–Whitney U-test was used to identify significant differences between two independent means. The estimated minimum number of participants was 34 (23 non-sarcopenia and 11 sarcopenia). Considering the potential for dropout, 38 participants (23 non-sarcopenia and 15 sarcopenia) were recruited. As a result, a total of 38 participants were enrolled. Participants were asked to maintain their normal dietary habits and refrain from vigorous physical activity the day before and immediately before the experiment. The participants’ demographic data included age, sex, height, body mass, body mass index (BMI), HbA1c level, duration of type 2 diabetes, grip strength, and skeletal muscle mass index (SMI).

The experimental protocol was approved by the Ethical Review Board at the Ethics Committee of the Kagawa University Faculty of Medicine (approval number: 2021-155). All participants provided written informed consent. The study complied with the principles of the Declaration of Helsinki.

### Definition of sarcopenia

Although several criteria have been proposed to define sarcopenia, only the recently reported AWGS 2019 criteria were used to classify participants into different groups in this study. The SMI was measured using BIA with a bioelectrical impedance data acquisition system (Inbody 770; Biospace Co., Ltd., Seoul, Korea). Low muscle mass was defined as an SMI value of < 7.0 kg/m^2^ in males and < 5.7 kg/m^2^ in females. Low muscle strength was defined as a handgrip strength of < 28 kg in males and < 18 kg in females. The criterion for low physical performance was a 6-m walking speed of < 1.0 m/s. Sarcopenia was defined as “low muscle mass + low muscle strength or low physical performance,” and severe sarcopenia was defined as “low muscle mass + low muscle strength and low physical performance.”

### Gait measurement

Gait measurements were obtained in a room with a straight 10-m path on which the participants could walk. During the experiment, all participants wore standardized clothing (sleeveless shirt and pants) provided by the experimenters, with clothing size selected by the participants. During gait measurement, participants were asked to walk barefoot at a comfortable, self-selected speed, and 3D positional data were obtained using reflective markers, a 10-camera, and a 3D motion capture system (MAC3D, Motion Analysis Corporation, Rohnert Park, California, USA) with a 200 Hz sampling frequency. A total of 57 infrared reflective markers were attached according to Visual 3D software (HAS-Motion Inc., Kingston, ON, Canada) guidelines. Simultaneously, ground reaction forces (GRFs) were obtained using four force plates (BP400600-2000, AMTI, Watertown, MA, USA) sampled at 2000 Hz. Marker positions were recorded with the participants standing stationary prior to the walking trials. Participants practiced walking to ensure a natural gait, followed by recordings of five successful trials per leg, in which each participant stepped correctly on a force plate. Based on the data obtained for each participant, the median, first, and third quartiles of spatiotemporal gait parameters and pelvis, hip, knee, and ankle joint angles were calculated at each time point.

### Data analysis

Raw motion and GRF data were digitally filtered using a zero-lag, fourth-order, low-pass Butterworth filter with cut-off frequencies of 10 Hz for positional data and 56 Hz for GRF data, based on a previous study^14^. The angles of the pelvis, hip, knee, and ankle joints during one gait cycle were calculated for the x axis (flexion–extension), y axis (abduction–adduction), and z axis (internal–external rotation) using a Cardan sequence of rotations (X-Y-Z) from the trajectories measured in each trial. Based on a previous study^15^, the angles were time-normalized using the gait cycle duration determined from the force plate data and divided into 101 data points ranging from 0 to 100%. Central tendencies (median and first and third quartiles) of walking speed, stride length, step width, stride time, stance time, swing time, double support time, and cadence were also determined to help understand gait characteristics. Low-pass filtering, variable calculation (i.e., spatiotemporal gait parameters and joint angles), and time normalization were performed using Visual 3D software. Walking speed, stride length, and step width were normalized and compared with reference to the non-dimensional normalization method proposed by Pinzone et al.^16^, as expressed in the following formula:

$$\:\:\:\:\:\:\:\:\:\:\:Normalized\:walking\:speed=\frac{walking\:speed}{\sqrt{g{h}_{0}}}$$

$$\:\:\:\:\:\:\:\:\:\:\:Normalized\:stride\:length\:or\:step\:width=\frac{stride\:length\:or\:step\:width}{{h}_{0}}$$

$$\:\:\:\:\:\:\:\:\:\:\:g\:is\:the\:acceleration\:of\:gravity\:(=9.81\:m/{s}^{2}),\:{h}_{0}\:is\:body\:height$$

Statistical analyses were performed using Statistical Package for the Social Sciences version 28.0 (SPSS Inc., Chicago, IL, USA). The Chi-square test was used to compare sex in non-sarcopenic and sarcopenic participants. The Mann–Whitney U tests were used to analyze the differences in other demographic data and spatiotemporal gait parameters of the participants. Effect sizes (ES*_r*) for the Mann-Whitney U test were calculated using the following formula:

$$\:ES\_r=z/\sqrt{N}$$

$$\:where\:z\:is\:the\:standardized\:test\:statistic,\:and\:N\:is\:is\:the\:total\:number\:of\:patients$$

Differences in range of motion (ROM; maximum angle minus minimum angle) of the pelvis, hip, knee, and ankle joint angles on the sagittal, frontal, and horizontal planes in the gait cycle between participants with and without sarcopenia were evaluated using ANCOVA, with gait speed included as a covariate. For comparisons where significant differences were observed (*p* < 0.05), effect sizes were evaluated using partial eta squared ($\:{\eta\:}_{p}^{2}$) and Cohen’s *f*. Cohen’s *f* was calculated from $\:{\eta\:}_{p}^{2}$​ using in the following formula:

$$\:\:f=\sqrt{{\eta\:}_{p}^{2}/(1-{\eta\:}_{p}^{2})}$$

The correlations between HbA1c levels, duration of type 2 diabetes, demographic data, and spatiotemporal gait parameters were analyzed using Spearman’s rank correlation coefficient. Differences in HbA1c levels and duration of type 2 diabetes by sex were analyzed using the Mann–Whitney U test. Statistical significance was set at *p* < 0.05.

## Results

The participants’ demographic data and spatiotemporal gait parameters are presented in Table 1. According to the criteria established by the AWGS 2019, among the 38 participants with type 2 diabetes, 15 were categorized as having sarcopenia (five of whom had severe sarcopenia) and 23 were classified as non-sarcopenic. Comparison of participants with and without sarcopenia showed significant differences in grip strength (*p* = 0.007, ES*_r* = − 0.429) and SMI (*p* = 0.006, ES*_r* = − 0.442); however, no significant differences were observed in age, sex, height, body mass, BMI, HbA1c level, or duration of type 2 diabetes mellitus. Regarding spatiotemporal gait parameters, participants with sarcopenia demonstrated significantly slower walking speed (*p* < 0.001, ES*_r* = − 0.632), shorter stride length (*p* = 0.003, ES*_r* = − 0.477), longer stride time (*p* = 0.004, ES*_r* = 0.461), longer stance time (*p* = 0.011, ES*_r* = 0.408), longer double support time (*p* = 0.048, ES*_r* = − 0.322), and lower cadence (*p* < 0.001, ES*_r* = − 0.540) than non-sarcopenic participants. However, no significant differences were observed in step width or swing time. In addition, no correlations exist between HbA1c levels or the duration of type 2 diabetes with demographic data or spatiotemporal gait parameters (Table 2).

**Table 1:** Demographic data and Spatiotemporal gait parameters of participants with and without sarcopenia.

Variables	Participants without sarcopenia (*n* = 23)		Participants with sarcopenia (*n* = 15)	*p*-value	Effect size
	(Median [Interquartile range])		(Median [Interquartile range])		(ES_r)
Demographic data					
Age (years)	74.0 [71.0–79.0]		77.0 [71.0–81.0]	0.460	0.121
Sex (male/female)	13/10		11/4	0.294	−0.170
Body height (m)	1.60 [1.56–1.66]		1.60 [1.54–1.65]	0.836	−0.034
Body mass (kg)	61.7 [55.3–71.4]		54.0 [51.7–59.1]	0.068	−0.298
BMI (kg/m^2^)	24.1 [22.0–26.5]		21.6 [20.2–24.5]	0.083	−0.283
HbA1c levels (%)	7.1 [6.6–7.4]		7.2 [6.8–7.8]	0.616	0.085
HbA1c levels (mmol/mol)	54 [49–57]		55 [51–62]	0.616	0.085
Duration of T2D (years)	14.1 [5.0–20.6]		7.2 [6.75–7.8]	0.286	0.174
Grip strength (kg)	27.7 [21.1–34.2]		21.6 [16.9–23.1]	0.007*	−0.429
SMI (kg/m^2^)	7.20 [6.35–7.75]		6.30 [5.80–6.50]	0.006*	−0.442
Sarcopenia criteria					
Sarcopenia (n)	0		10		
Severe sarcopenia (n)	0		5		
Spatiotemporal gait parameters					
Walking speed (m/s)	1.26 [1.15–1.32]		1.02 [0.85–1.08]	< 0.001*	−0.613
Normalized walking speed (dimensionless)	0.31 [0.29–0.34]		0.26 [0.21–0.27]	< 0.001*	−0.632
Stride length (m)	1.21 [1.15–1.27]		1.05 [0.96–1.16]	0.004*	−0.453
Normalized stride length (dimensionless)	0.75 [0.71–0.79]		0.68 [0.58–0.71]	0.003*	−0.477
Step width (m)	0.11 [0.09–0.13]		0.12 [0.10–0.14]	0.401	0.370
Normalized step width (dimensionless)	0.07 [0.06–0.08]		0.10 [0.08–0.32]	0.058	0.292
Stride time (s)	0.97 [0.94–1.03]		1.05 [1.01–1.12]	0.004*	0.461
Stance time (s)	0.56 [0.53–0.60]		0.62 [0.58–0.69]	0.011*	0.408
Swing time (s)	0.41 [0.39–0.43]		0.43 [0.41–0.44]	0.095	0.273
Double support time (s)	0.17 [0.15–0.20]		0.21 [0.17–0.25]	0.048*	0.322
Cadence (steps/min)	125.8 [119.0–130.6]		111.7 [105.7–116.8]	< 0.001*	−0.540

*T2DM* type 2 diabetes, *SMI* skeletal muscle mass index.

*p < 0.05.

**Table 2:** Relationship between diabetes-related indicators and demographic data and Spatiotemporal gait parameters.

Variables	Correlation with HbA1c levels			Correlation with duration of type 2 diabetes		
	HbA1c level (%)	*r*	*p*-value	Duration of T2D (years)	*r*	*p*-value
Demographic data						
Age		−0.221	0.183		0.183	0.272
Sex			0.308			0.489
Male	7.1 [6.7–7.5]			15.5 [6.3–20.3]		
Female	7.2 [6.7–7.7]			17.5 [12.1–23.7]		
Body height		0.197	0.235		−0.105	0.531
Body mass		0.024	0.887		−0.307	0.061
BMI		−0.173	0.298		−0.183	0.343
Grip strength		0.016	0.923		−0.105	0.531
SMI		−0.116	0.488		−0.237	0.152
Spatiotemporal gait parameters						
Walking speed		−0.215	0.194		−0.226	0.173
Normalized walking speed		−0.229	0.167		−0.201	0.227
Stride length		−0.097	0.562		−0.241	0.146
Normalized stride length		−0.177	0.288		−0.208	0.210
Step width		−0.242	0.143		0.128	0.442
Normalized step width		−0.248	0.133		0.161	0.333
Stride time		0.218	0.188		0.024	0.885
Stance time		0.197	0.235		−0.002	0.990
Swing time		0.198	0.232		0.184	0.269
Double support time		0.113	0.498		−0.049	0.770
Cadence		−0.221	0.182		0.006	0.973

*SMI* skeletal muscle mass index, *T2DM* type 2 diabetes.

**Fig. 1:**
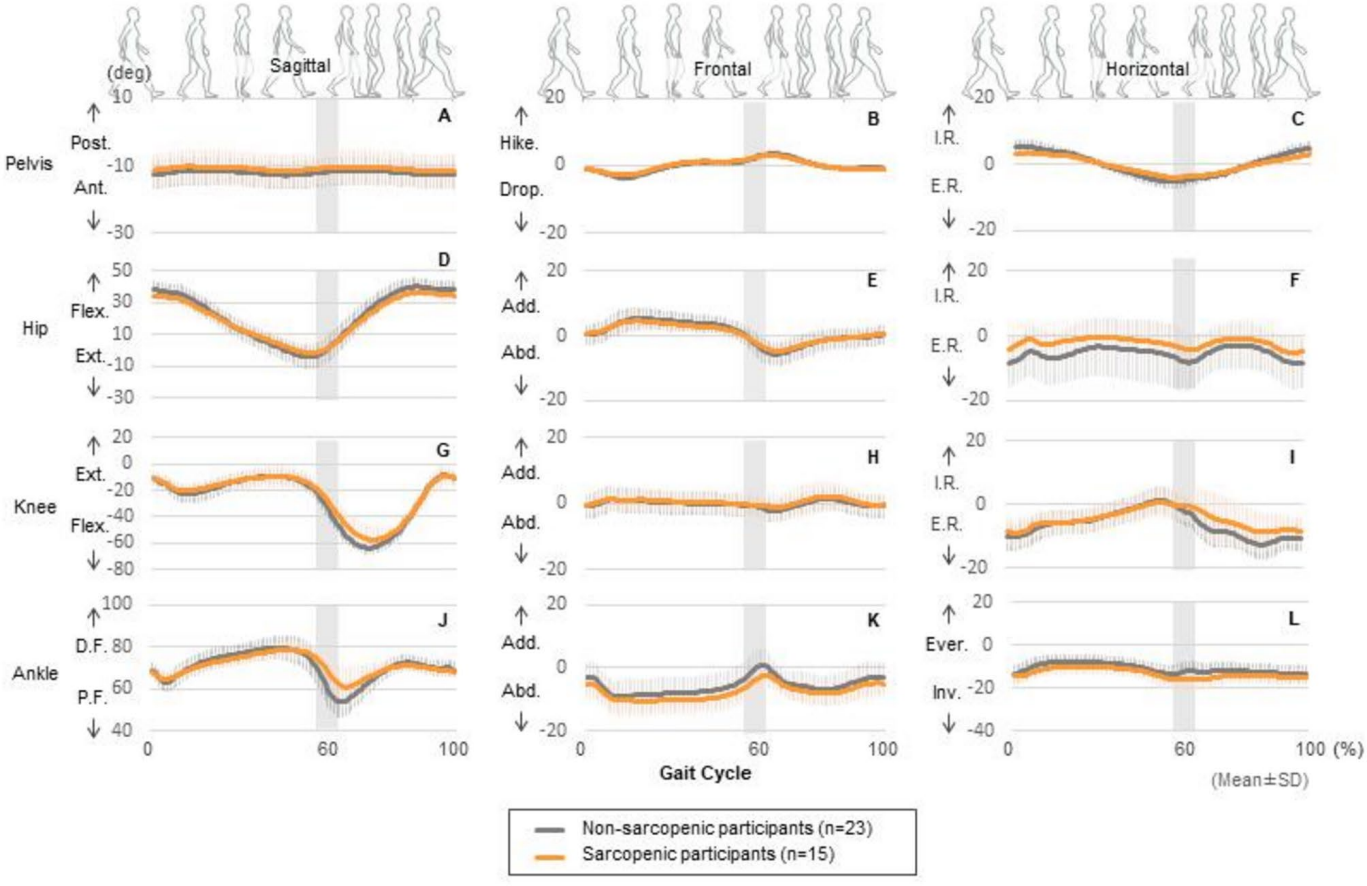
Waveforms of central tendency. Data are expressed as means ± standard deviation (SD). The gray highlighted area indicates the instance of the toe off (the transition from the stance phase to the swing phase). This area has a certain width because the stance phase was not separated from the swing phase in the time-normalization procedure. The joint motion waveforms of the non-sarcopenic and sarcopenic participants are shown in gray and orange, respectively. *Post.* posterior tilt, *Ant.* anterior tilt, *Hike.* pelvic hike, *Drop.* pelvic drop, *I.R.* internal rotation, *E.R.* external rotation, *Flex.* flexion, *Ext.* extension, *Add.* adduction, *Abd.* abduction, *D.F.* dorsi-flexion, *P.F.* plantar flexion, *Ever.* eversion, *Inv.* inversion, *deg.* degree.

**Fig. 2:**
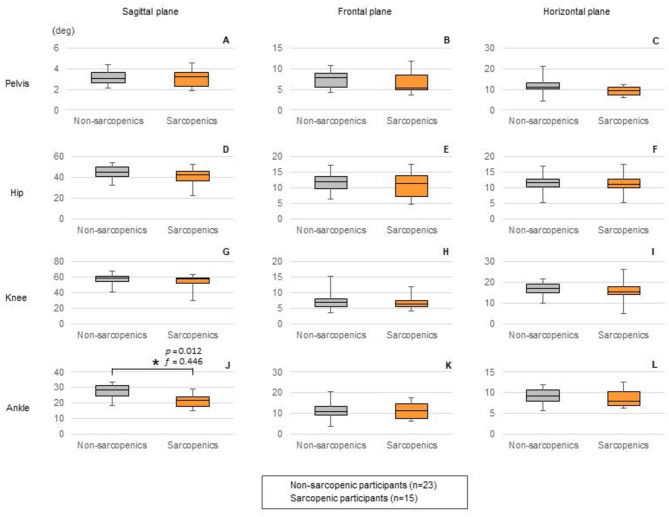
Comparison of joint ranges of motion during the gait cycle. Data are presented as medians and interquartile ranges. The whiskers indicate the minimum and maximum values. The ranges of motion for the participants with and without sarcopenia are shown using orange and gray box plots, respectively. The asterisks indicate significant differences between the participants with and without sarcopenia (*p < 0.05). *deg.* degree.

The kinematic waveforms of the pelvis, hip, knee, and ankle joint angles in the sagittal, frontal, and horizontal planes are illustrated in Fig. 1. Each waveform was divided into 101 time frames for one gait cycle, starting from the moment of heel contact. To account for variations in the ratio of stance time to swing time among participants, a gray bar indicated the estimated toe-off phase, delimiting the stance phase before it and the swing phase after it. Comparisons of ROM for each joint from these joint kinematic waveforms during the gait cycle revealed that participants with sarcopenia had a significantly smaller ROM for ankle joint plantar dorsiflexion (*p* = 0.012, $\:{\eta\:}_{p}^{2}$ = 0.166, *f* = 0.446) (Fig. 2).

## Discussion

This study aimed to clarify the differences in gait characteristics between older patients with type 2 diabetes, with and without sarcopenia. The findings revealed that patients with type 2 diabetes and sarcopenia had lower grip strengths, decreased SMIs, and slower walking speeds than those with their counterparts without sarcopenia. Furthermore, comparison of spatiotemporal parameters of gait revealed that these patients had shorter stride lengths, longer stride times, stance times and double support times, and lower cadence. Decreased range of ankle joint plantar dorsiflexion angles during the gait cycle were also observed in patients with sarcopenia and type 2 diabetes. Previous studies have analyzed spatiotemporal gait parameters in individuals with sarcopenia^12,13,17,18^ and joint ROM during walking in older adults^19^. However, no studies have compared joint ROM during walking between individuals with and without sarcopenia or specifically examined joint ROM during walking in patients with type 2 diabetes. Therefore, this study is the first to reveal the effect of sarcopenia on spatiotemporal and kinematic parameters of gait in patients with type 2 diabetes.

The development of sarcopenia is influenced by age-related physiological changes and the pathophysiology of type 2 diabetes. Older patients with type 2 diabetes reportedly experience muscle mass reduction^20^ and decreased muscle strength^21^ compared with their healthy counterparts. The mechanisms by which type 2 diabetes induce sarcopenia include insulin deficiency, hyperglycemia, and vascular and neuropathic complications. Protein synthesis may be one of the factors explaining muscle atrophy observed in patients with type 2 diabetes. A cross-sectional study of 13,644 participants using data from the National Health and Nutrition Examination Survey showed a negative correlation between insulin resistance, assessed by the homeostasis model assessment for insulin resistance, and muscle mass^22^. Furthermore, a cross-sectional study conducted in Taiwan that analyzed 844 older individuals found that, alongside reduced physical activity, insulin resistance was associated with a decrease in skeletal muscle mass^23^. Barzilay et al.^24^ reported that insulin resistance was associated with decreased muscle strength and walking speed. Epidemiological studies have suggested a relationship between hyperglycemia and sarcopenia. In a cross-sectional study involving 746 older patients with type 2 diabetes, an increased prevalence of sarcopenia was observed among patients with higher HbA1c levels. In the group with HbA1c levels of ≥ 8.0%, the risk of skeletal muscle mass reduction (SMI: <7.0 kg/m^2^ for males, < 5.7 kg/m^2^ for females) was 5.35 times higher than that in the group with HbA1c levels of ≤ 6.5%^25^. Furthermore, longitudinal studies have revealed significant declines in muscle strength and physical activity capacity in individuals stratified by HbA1c levels, particularly in the hyperglycemic group^26^. However, when comparing between the non-sarcopenic and sarcopenic participants in this study, no significant differences were observed in HbA1c levels or duration of type 2 diabetes. This discrepancy may be attributed to the fact that the HbA1c value during the gait test may not fully reflect glycemic control throughout the disease period, as this study was not a time-limited clinical investigation. This study aimed to analyze the gait function in older patients with sarcopenia and type 2 diabetes. Several time-limited interventions have indicated that glycemic control is crucial to the development and progression of sarcopenia. Additionally, vascular impairment^27,28^ and neuropathy^29^ associated with type 2 diabetes may contribute to the development of sarcopenia. However, individuals with severe vascular or neuropathic impairments were excluded from this study, making it unlikely that these factors significantly affected the results. Although the findings of this study did not reveal the specific impact of type 2 diabetes on gait characteristics, some gait characteristics specific to patients with type 2 diabetes are potentially present in the results.

The prevention and management of sarcopenia could play a crucial role in enhancing glycemic control in patients with type 2 diabetes. However, research evaluating the differences in gait characteristics in patients with type 2 diabetes, with or without sarcopenia, is lacking. In this study, HbA1c levels and the duration of type 2 diabetes did not reveal any correlation with spatiotemporal gait parameters. Future studies should explore gait characteristics in relation to glycemic control over a longer period of time to determine the effects of glycemic control on gait characteristics. Fan et al.^17^ demonstrated that older adults with low skeletal muscle mass exhibit shorter step and stride lengths than those with normal skeletal muscle mass. The decreased walking speed among individuals with sarcopenia may be attributed to shortened stride length^18^. Additionally, gait analyses in older individuals have reported age-related decreases in walking speed, shortened stride length, widened step width, increased double support time, and decreased cadence (prolonged stride time)^30^. The results of this study, which showed decreased walking speeds and cadence and shortened stride lengths and prolonged stride times, stance times and double support times among participants with sarcopenia, generally support the findings of previous studies.

Few previous studies have investigated joint ROM during the gait cycle in individuals with or without sarcopenia. In a systematic review of gait patterns in older adults, moderate evidence indicated that they exhibited reduced pelvic rotation range during comfortable walking^31^. Considering that older adults often experience significant reductions in muscle mass and strength compared with younger individuals^32^, as well as weakness in trunk muscle strength^32–34^, the decreased pelvic rotation range observed among participants with sarcopenia (Supplemental data), may be attributed to the decline in muscle strength, particularly when walking speed is not accounted for. Judge et al.^19^ reported a significant decrease in the maximum ankle joint dorsiflexion angle during the terminal stance phase in older individuals compared with younger individuals, attributing this decrease to reduced eccentric contraction strength of the gastrocnemius and soleus muscles. The decrease in the maximum ankle dorsiflexion angle during the terminal stance phase, as demonstrated in our waveforms, suggests that similar ankle joint movement characteristics occur in patients with sarcopenia and type 2 diabetes. However, no previous study has investigated the decrease in ankle plantar dorsiflexion ROM during gait in individuals with sarcopenia. Therefore, future research should examine the relationship between these joint ROMs and muscle strength and include biomechanical analyses of gait characteristics.

The results of this study showed that the gait characteristics of older patients with type 2 diabetes were decreased walking speed and cadence, shorter stride lengths, longer stride, stance and double support times, and decreased ROM of ankle plantar dorsiflexion. By evaluating gait based on these endpoints, it may be possible to objectively evaluate the impact of sarcopenia on gait in patients with type 2 diabetes. Moreover, incorporating gait analysis tools, such as AI posture-estimation systems that utilize mobile or tablet devices, into clinical practice along with gait assessments conducted by physical therapists may prove beneficial.

This study had some limitations. First, the influence of mild vascular or neurological impairments could not be completely excluded. Second, we did not analyze participants separately based on sex. As the diagnostic criteria for sarcopenia vary according to sex, we were unable to assess the influence of sex on the impact of sarcopenia on physical function. Older men generally exhibit faster walking speeds, longer stride lengths, and lower cadence than women, suggesting potential differences in walking patterns between older men and women^35,36^. Third, this study did not compare older individuals without diabetes to older patients with type 2 diabetes; thus, we could not examine the impact of type 2 diabetes on gait characteristics. Fourth, the relationship between glycemic control using factors such as HbA1c levels and gait parameters was not evaluated. Evaluation of the relationships between glycemic control and related parameters, such as insulin resistance, and gait parameters, including walking speed, is a subject for future studies. Finally, the small sample size in this study might limit the generalizability of the findings.

This study compared the spatiotemporal and kinematic parameters of gait between patients with type 2 diabetes with and without sarcopenia. Patients with type 2 diabetes and sarcopenia had slower walking speeds, shorter stride lengths, longer stride, stance and double support times, and lower cadence than their counterparts without sarcopenia. In addition, decreased range of ankle joint plantar dorsiflexion angles during the gait cycle were also observed in patients with type 2 diabetes and sarcopenia. Our findings indicate that patients with type 2 diabetes and sarcopenia exhibit not only decreased walking speed but also abnormalities in walking rhythm and reduced joint mobility in the gait cycle. Assessment of these gait characteristics may be an indicator of sarcopenia in older patients with type 2 diabetes. In addition to contributing new knowledge to the field, this may have practical implications for sarcopenia detection in clinical settings.

## Electronic supplementary material

Below is the link to the electronic supplementary material.

Supplementary Material 1

## Data Availability

The datasets generated during and/or analyzed during the current study are available from the corresponding author on reasonable request.
