# Supplementary material for: Spatiotemporal and kinematic gait characteristics in older patients with type 2 diabetes mellitus with and without sarcopenia
Source: Sci Rep. 2025 May 27;15:18000. doi: 10.1038/s41598-025-00205-0 (PMC12116902; doi:10.1038/s41598-025-00205-0)
Supplement: Supplementary file 1 — Supplementary Material 1 [file 41598_2025_205_MOESM1_ESM.docx]

**Supplemental Data**

Supplemental Table 1. Data on joint ranges of motion during the gait cycle. The data are presented as medians and interquartile ranges. Mann–Whitney U tests were used to analyze the differences in joint ranges of motion between participants with and without sarcopenia. Additionally, effect sizes (*ES_r*) were calculated to validate the results of the Mann–Whitney U test. Statistical significance was set at p < 0.05.

| Variables | Participants without sarcopenia  (n=23) | Participants with sarcopenia  (n=15) | *p*-value | Effect sizes  (*ES_r*) |
| --- | --- | --- | --- | --- |
|  | (Median [Interquartile range]) | (Median [Interquartile range]) |  |  |
| Pelvis |  |  |  |  |
| x axis | 3.1 [2.6–3.6] | 3.3 [2.3–3.6] | 0.906 | −0.019 |
| y axis | 7.9 [5.6–8.9] | 5.4 [4.8–8.5] | 0.300 | −0.172 |
| z axis | 11.1 [10.2–13.1] | 9.3 [7.5–11.0] | 0.011* | −0.409 |
| Hip |  |  |  |  |
| x axis | 44.4 [41.0–50.2] | 42.2 [36.2–45.6] | 0.078 | −0.286 |
| y axis | 11.9 [9.6–13.6] | 11.3 [7.1–13.8] | 0.516 | −0.107 |
| z axis | 11.5 [10.2–12.7] | 11.1 [10.1–12.7] | 0.836 | −0.036 |
| Knee |  |  |  |  |
| x axis | 58.6 [54.7–61.4] | 57.4 [52.8–58.8] | 0.202 | −0.211 |
| y axis | 6.8 [5.6–8.1] | 6.4 [5.4–7.5] | 0.575 | −0.092 |
| z axis | 17.0 [15.1–19.3] | 15.6 [14.3–18.0] | 0.442 | −0.128 |
| Ankle |  |  |  |  |
| x axis | 28.5 [24.4–31.2] | 21.7 [18.1–23.9] | <0.001* | −0.584 |
| y axis | 11.0 [9.3–13.4] | 11.1 [7.4–14.5] | 0.595 | −0.09 |
| z axis | 9.2 [8.0–10.6] | 8.0 [6.8–10.3] | 0.595 | −0.087 |
| x axis: flexion–extension), y axis: abduction–adduction, z axis: internal–external rotation | | | | |
